# Shockwave Lithotripsy for Calcific Mitral Valve Stenosis: A Promising Technology

**DOI:** 10.1016/j.jscai.2022.100549

**Published:** 2023-01-10

**Authors:** Kinan Kassar, Adnan Khalif, David Lasorda

**Affiliations:** Allegheny General Hospital, Department of Cardiology, Pittsburgh, Pennsylvania

**Keywords:** calcific mitral stenosis, lithotripsy, valvuloplasty

Percutaneous balloon mitral valvuloplasty is the treatment of choice for minimally calcified mitral stenosis with a low Wilkins score^1^; however, heavily calcified valves continue to present a management challenge, especially in high surgical risk patients.

## Case presentation

An 84-year-old woman with New York Heart Association class III heart failure symptoms was found to have severe calcific mitral valve stenosis.

Transesophageal echocardiogram showed significant mitral annular calcification extending mainly on the lateral side of the posterior leaflet, leading to a complete fixation of the leaflet in a prolapse position into the left atrium (Supplemental Video 1). The mean mitral valve pressure gradient (mPG) was 8 mm Hg at a heart rate (HR) of 58 bpm, and the 3-dimensional mitral valve area (MVA) was 0.6 cm^2^ with mild mitral regurgitation.

Given her advanced age and frailty, the patient was deemed high risk for surgical mitral valve replacement by our multidisciplinary heart team. She was screened for possible transcatheter mitral valve in mitral annular calcification; however, her MVA was extremely small.

There was a significant concern regarding the efficacy of balloon valvuloplasty because of the thickness and density of the calcium burden on the mitral valve. The Wilkins score was 14. Given the success described in multiple initial reports with mitral valve Shockwave lithotripsy balloon valvuloplasty, we decided to use the technology to debulk the valvular calcium.

## Procedure description

The procedure was performed under general anesthesia and an activated clotting time of >250 seconds.

A Swan-Ganz catheter was inserted, and a pigtail catheter was placed in the left ventricle for hemodynamic monitoring. A cerebral embolic protection device (Sentinel; Boston Scientific) and a temporary pacemaker wire were also used.

An 18F DrySeal sheath catheter (Gore Medical) was introduced into the right common femoral vein with an 8.5F steerable Mobicath catheter (Biosense Webster) for a targeted transseptal puncture.

The mitral valve was crossed with Judkins wire, which was exchanged with a Safari wire (Boston Scientific) over a pigtail catheter.

The initial left atrium–left ventricle mPG was 6.6 mm Hg with a cardiac output of 2.6 L/min at an HR of 44 bpm. The Gorlin-formula derived MVA was 1.1 cm^2^.

A 14 × 60-mm XXL vascular balloon (Boston Scientific) was used to dilate the intra-atrial septum over Safari wire followed by the advancement of a 45° angulated 12F Amplatzer Trevisio sheath (Abbott).

A 7F multipurpose guiding catheter was introduced through the sheath, and 2 IronMan 0.014-inch wires were advanced into the left ventricle cavity. The multipurpose guide was removed before inserting two 8.0 × 60-mm peripheral Shockwave lithotripsy balloons (Shockwave Medical) over the wires.

**Figure 1:**
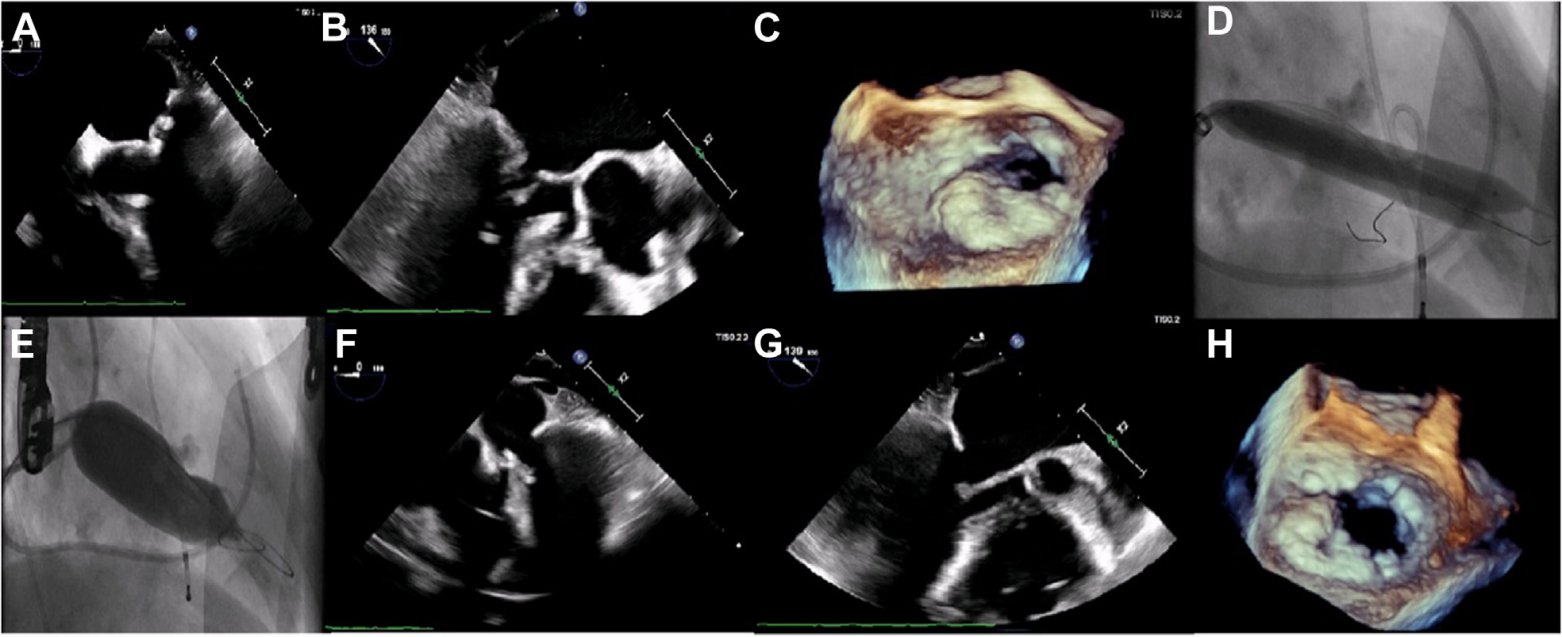
(**A, B,C**) Preprocedure transesophageal echocardiogram of severely calcified stenotic mitral valve with significant posterior leaflet calcification and decreased mobility (Supplemental Video 1). (**D**) Mitral valvuloplasty with 2 simultaneous 8.00 × 60-mm Shockwave lithotripsy balloons. (**E**) Mitral valvuloplasty with 25 × 40-mm True Dilation balloon (Supplemental Video 2). (**F, G, H**) Postprocedure transesophageal echocardiogram showing improvement in mitral valve area and leaflet mobility (Supplemental Video 3).

We delivered 4 lithotripsy runs of 30 pulses/15 s for a total of 120 pulses. Shockwave balloons were inflated to 4 atmospheres during the first 2 runs and to 6 atmospheres for the second 2 runs (Figure 1). The patient was allowed a few minutes to recover her hemodynamics in between the Shockwave runs.

IronMan wires were exchanged with a Safari wire over the multipurpose catheter; further, a 25 × 45-mm True Dilatation balloon (Bard) was used for mitral valvuloplasty (Supplemental Video 2).

**Supplemental Figure S1:**
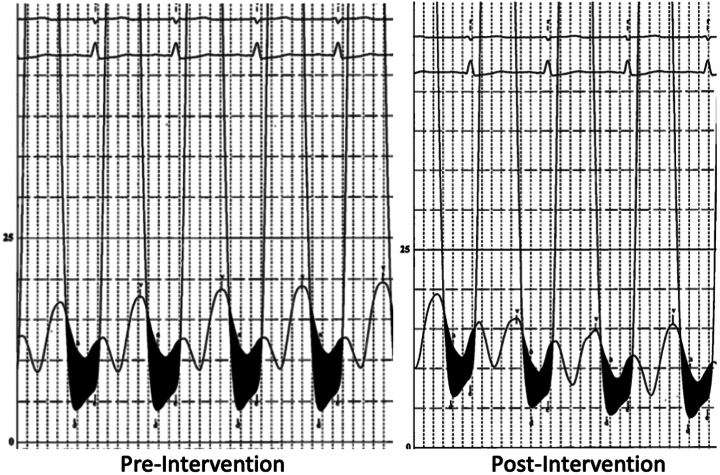
Simultaneous LA-LV pressure tracings showing normal and similar left ventricular end diastolic pressure pre and post intervention with a decrease in LA pressure after valvuloplasty.

Repeat hemodynamic measurements showed an mPG of 4 mm Hg with cardiac output of 5.4 L/min at an HR of 78 bpm (Supplemental Figure S1). The Gorlin formula-derived MVA was 3.1 cm^2^. Transesophageal echocardiogram 3-dimensional planimetry MVA was 1.5 cm^2^ with an mPG of 4 mm Hg at an HR of 66 bpm with mild-to-moderate mitral regurgitation after valvuloplasty (Supplemental Video 3).

The patient was neurologically intact on extubation with no debris captured in the Sentinel device. She was discharged home the following day.

## Discussion

Data on the utility of Shockwave lithotripsy of calcific mitral valve to facilitate percutaneous balloon valvuloplasty are scarce.^2^, ^3^, ^4^ A report of a limited number of cases showed a trend toward lower postprocedure invasive mPG and higher rates of procedural success.^5^ It is hypothesized that similar to the coronary and peripheral vascular application of Shockwave lithotripsy, fracking thick valvular calcium allows more expansion of valvuloplasty balloon and renders the calcified mitral leaflet tips pliable to prevent significant mitral regurgitation after the intervention. We report the first use of the new 8-mm Shockwave balloons (compared with 7-mm balloons) that have the capability of delivering 2 shocks/s, which helps shorten the occlusive time of the mitral valve. The length of the balloons allowed stability, and pacing was not needed. Our case demonstrates the safety and efficacy of using the technology to manage calcific mitral stenosis. We look forward to more advancements in the technology and lithotripsy balloon design to allow use of 1 adequately sized balloon in the future.

## Supplementary material

Video 1 2D and 3D transesophageal echocardiograms of heavily calcified and stenosed mitral valve prior to valvuloplasty.

Video 2 Shockwave lithotripsy of mitral valve with 2 shockwave balloons 8.0 × 60 mm over 2 IronMan 0.014’ wires followed by mitral valvuloplasty with 25 × 45 mm True Dilation balloon over Safari wire.

Video 3 2D and 3D transesophageal echocardiogram showing significant improvement in mitral valve area after valvuloplasty with only mild to moderate mitral regurgitation.
